# Hospitalization requiring intensive care unit due to SARS-CoV-2 infection correlated with IgM depression and IgG elevation

**DOI:** 10.2144/fsoa-2021-0126

**Published:** 2022-02-02

**Authors:** Sara Tamizuddin, Jason Cham, Yasamin Ghiasi, Luis Borroto, Cindy Cao, Natalia Orendain, Michael M Quigley, Laura J Nicholson, Amitabh C Pandey

**Affiliations:** ^1^Department of Medicine, Scripps Clinic/Scripps Green Hospital, La Jolla, CA 92037, USA; ^2^Scripps Research Translational Institute, The Scripps Research Institute, La Jolla, CA 92037, USA; ^3^Ponce Health School of Medicine & Health Sciences, Ponce, 00731, Puerto Rico; ^4^Scripps Whittier Diabetes Institute, Scripps Hub Academic Research Core, La Jolla, CA 92037, USA; ^5^Department of Pathology, Scripps Clinic/Scripps Green Hospital, La Jolla, CA 92037, USA; ^6^Division of Cardiology, Scripps Clinic, La Jolla, CA 92037, USA

**Keywords:** antibody response, COVID-19, ICU, immunology, infectious disease, SARS-CoV-2, spike protein

## Abstract

**Aim:** This study investigated the humoral response against SARS-CoV-2 in patients needing intensive care unit (ICU) care compared with those on general medicine wards. **Materials & methods:** The authors retrospectively reviewed 113 hospitalized patients with COVID-19. They assessed antibody response against five SARS-CoV-2 epitopes at 6–14 days post symptom onset in these patients. **Results:** Patients with ICU admissions had decreased anti-nucleocapsid immunoglobulin (Ig)M and increased anti-spike IgG compared with patients not requiring the ICU. IgG levels were positively correlated with length of stay. **Conclusion:** Higher levels of IgG against the spike protein correlate with COVID-19 disease severity and length of stay in hospitalized patients. This adds to the knowledge of biochemical response to clinical disease and may help predict ICU needs.

## Plain language summary

This study analyzed blood samples in patients hospitalized with COVID-19 at 6–14 days after their initial symptom onset. Those who needed care in the intensive care unit had lower levels of an early antibody (IgM) to a protein inside the virus and higher levels of a later antibody (IgG) to the spike protein on the outer viral membrane. Higher IgG levels were also associated with longer hospital stays.

The respiratory virus SARS-CoV-2 has caused unexpectedly high mortality and morbidity since the outbreak began. As of 1 November 2021, over 250 million confirmed cases and 5 million deaths have been reported worldwide due to COVID-19 [1]. This disease has led to a shortage of hospital beds, particularly in intensive care units (ICUs), and increasing pressure on staff and healthcare resources. The COVID-19 pandemic has launched multiple diagnostic platforms offering rapid data that have significant impact on patient care. Serologic testing for antibodies is an accepted modality for monitoring pathogen response, allowing the determination of prior infection and/or vaccination, but specific data allowing a precise understanding of humoral response to SARS-CoV-2 are still accumulating [2]. Pro-inflammatory markers may be used to track patients' disease progression, but increased understanding of antibody timing, class and level could help better stratify risk stratification, such as those who will require ICU level of care [3]. This is especially true with the emergence of SARS-CoV-2 variants, with the potential to increase hospitalizations.

The SARS-CoV-2 genome encodes the S protein, which mediates cellular infection and is divided into two subunits: S1 and S2. The S1 subunit contains the receptor binding domain (RBD), which facilitates viral entry into cells. The S2 subunit enables viral membrane and host cellular membrane fusion [4]. Given these critical properties, the S protein became the primary target for vaccination-induced immunity. Antibodies against this protein can be detected within 1 to 3 weeks of natural infection, and rapid antibody production (both immunoglobulin [Ig]M and G) occurs between 7 and 10 days from symptom onset [5,6].

This study aimed to characterize the antibody response in hospitalized COVID-19 patients to assess the relationships among antibody response, disease severity and patient outcome. As antibody testing becomes more prevalent, it is important to understand what serologic profiles may be expected in severe disease and if they may help guide management in the hospital.

## Materials & methods

The authors conducted a retrospective cohort study of hospitalized patients with COVID-19 from April to July 2020, prior to vaccine availability. The study was approved by the institutional review board. Included subjects were ≥18 years of age who had been admitted to the hospital for SARS-CoV-2 infection, confirmed by nasopharyngeal or oropharyngeal nucleic acid amplification testing on either the Panther Fusion (Hologic, MA, USA) or the Abbott ID Now platform (Abbott Laboratories, IL, USA).

Baseline comorbidities, hospital course and mortality were collected from the electronic medical record. Date of symptom onset was determined by retrospective chart review. Serum samples available between 6 and 14 days following symptom onset were assayed for the study. Patients were stratified by severity of disease, with less severe disease defined as hospitalization only on a general medical floor (“ward” group) and more severe disease defined as requiring ICU care at some point during hospitalization (“ICU” group). The requirements of ICU level of care included – but were not limited to – intubation, vasopressor support and continuous renal replacement therapy. Serologic assessment was done with the Maverick SARS-CoV-2 immunoassay (Genalyte, Inc., CA, USA), which was approved by US FDA Emergency Use Authorization in February 2020. This panel analyzes IgM and IgG response to five unique antigens from SARS-CoV-2 virus domains – nucleocapsid, spike S1 subunit, spike S1 RBD, spike S2 subunit and spike S1S2 protein. Controls included antigens from four less virulent species of coronavirus, influenza hemagglutinins, Middle East respiratory syndrome (MERS) virus and SARS-CoV-1. Briefly, 10 ul serum is placed in a plate array and baseline resonance is assessed. Detection of antibodies is based on photonic ring resonance, which detects changes in wavelength as antibodies bind to presented antigens. Results are reported in genalyte response units (GRUs). The assay reports an overall sensitivity of 98% and specificity of 100%, as good as or better than three other previously validated SARS-CoV-2 serologic assays [7].

Between-group baseline characteristics were compared using the Mann–Whitney test for continuous variables and chi-squared test for categorical variables. For the primary analysis, comparisons of continuous variables and categorical variables were performed using a one-way analysis of variance (ANOVA) or Kruskal–Wallis test. Correlation was tested using the Spearman rank correlation. R is the Spearman rank correlation coefficient. Statistical significance was declared at p-value ≤ 0.05, and no multiple testing adjustment was done. Analysis was done with the statistical computing software R (https://www.r-project.org/).

## Results

Sera from 113 patients hospitalized between April 2020 and July 2020 were analyzed for IgM and IgG response to SARS-CoV-2. Average age was 61 ± 16.7 years and average BMI was 32.4 ± 9.4. The majority of patients were Hispanic (62%) and female (58%), and comorbidities included hypertension (60%), diabetes (54%) and cancer history (3%), with 10% actively smoking. Two patients (2%) were pregnant. Comparisons between ward patients with no ICU needs (n = 58) and ICU patients who required ≥1 ICU day during hospitalization (n = 55) revealed no significant differences in background demographics or health characteristics (Tables 1 & 2). The ICU group had a significantly longer length of stay (mean 28 vs 9 days; p ≤ 0.0001) and had higher mortality (40% vs 7%; p ≤ 0.0001) (Table 1).

**Table 1.** Demographic characteristics of study sample (n = 113).

Characteristic	Observations, n (%)		p-value^†^
	ICU patients (n = 55)	Wards patients (n = 58)	
Age, years (M, SD)	63.29 (13.62)	59.21 (19.12)	0.153
Sex			
Male	18 (32.7)	30 (51.7)	0.136
Female	37 (67.3)	28 (48.3)	
Race and ethnicity			
White	7 (12.7)	11 (19.0)	0.508
Black	1 (1.8)	2 (3.4)	
Asian	4 (7.3)	4 (6.9)	
Hispanic	33 (60.0)	37 (63.8)	
Other	1 (1.8)	0 (0)	
Unknown	9 (16.4)	4 (6.9)	
BMI (M, SD)	34.50 (11.45)	30.42 (6.30)	0.181
Length of stay, days (M, SD)	28.40 (18.14)	8.98 (5.72)	<0.0001
ICU length of stay, days (M, SD)	20.93 (15.29)	0 (0)	<0.0001
From symptom to labs, days (M, SD)	8.78 (1.83)	8.88 (2.19)	0.963

† p-value significance is set at 0.05. p-values from Mann–Whitney test for continuous variables and chi-squared test for categorical variables.

ICU: Intensive care unit; M: Mean; SD: Standard deviation.

**Table 2.** Health characteristics of study sample (n = 113).

Characteristic	Observations, n (%)		p-value^†^
	ICU patients (n = 55)	Wards patients (n = 58)	
Hypertension	37 (67.3)	31 (53.4)	0.155
Diabetes mellitus	35 (63.6)	26 (44.8)	0.062
Nonhematologic cancer, on chemotherapy	0 (0)	1 (1.7)	>0.999
Nonhematologic cancer, not on chemotherapy	0 (0)	1 (1.7)	>0.999
Hematologic cancer, on chemotherapy	0 (0)	1 (1.7)	>0.999
Hematologic cancer, not on chemotherapy	0 (0)	0 (0)	–
Use of anti-hypertensive medications			
Calcium channel blocker	13 (23.6)	9 (15.5)	0.312
Beta blocker	14 (25.5)	13 (22.4)	0.739
ACE-inhibitor use	25 (45.5)	17 (29.3)	0.065
Diuretic	14 (25.5)	7 (12.1)	0.081
Central agonist	1 (1.8)	0 (0)	0.955
Vasodilator	3 (5.5)	1 (1.7)	0.533
Use of disease modifying anti-rheumatic drugs	2 (3.6)	0 (0)	0.213
Current smoker	4 (7.3)	7 (12.1)	0.131
Pregnant	0 (0)	2 (3.4)	0.499
Mortality	22 (40.0)	4 (6.9)	<0.0001

† p-value significance is set at 0.05. p-values from Mann–Whitney test for continuous variables and chi-squared test for categorical variables.

ACE: Angiotensin-converting enzyme; ICU: Intensive care unit; M: Mean; SD: Standard deviation.

**Figure 1.**
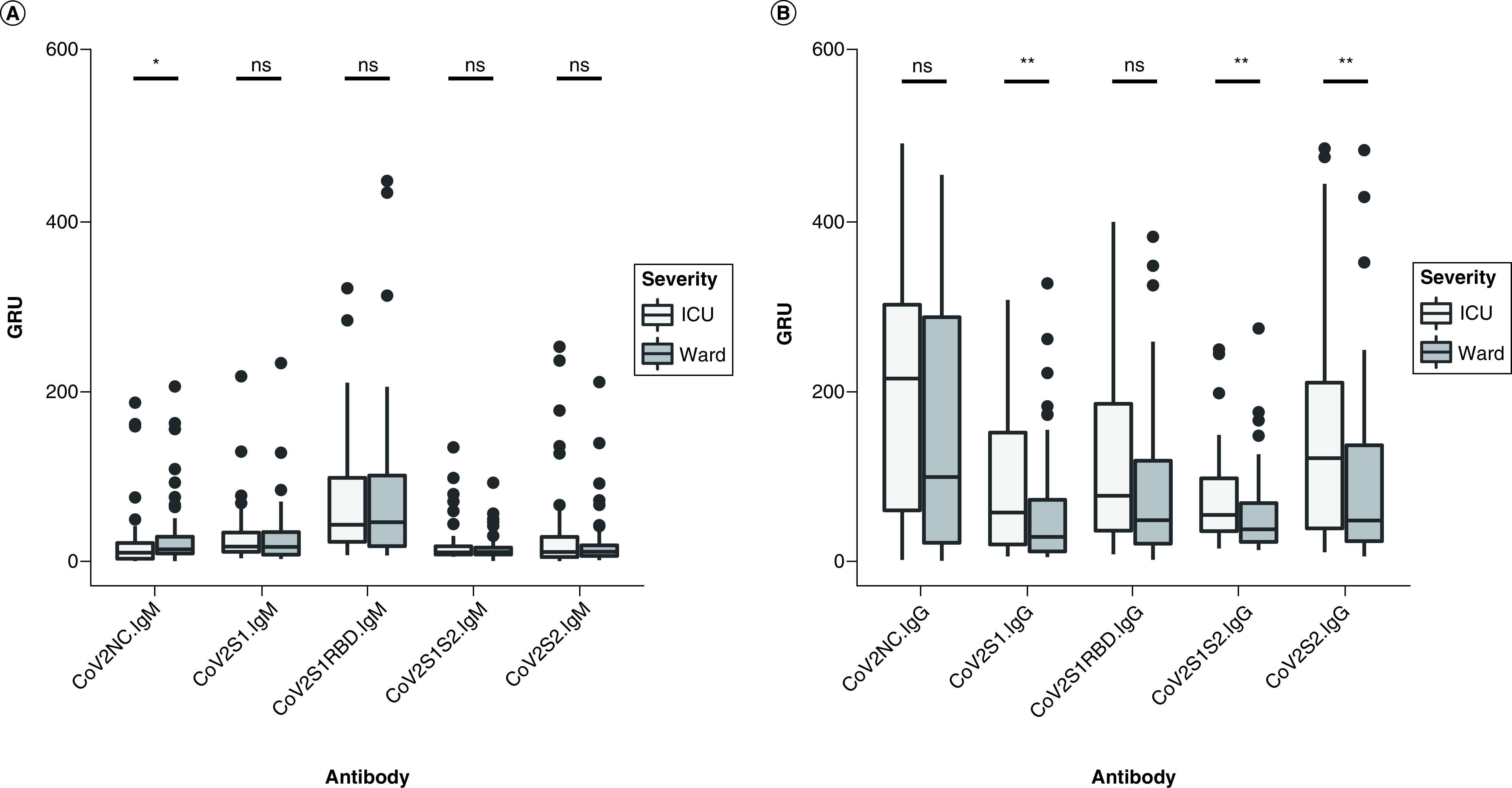
Immunoglobulin M and G in SARS-CoV-2 patients. **(A)** Antibody response to five SARS-CoV-2 epitopes in ICU patients versus general medical ward patients was assessed in GRUs for IgM and IgG. **(B)** Antibody response at a median of 8 days after symptom onset. Patients requiring ICU admission had lower levels of IgM against the nucleocapsid protein (CoV2NC.IgM) and higher levels of IgG against three out of four S protein domains (CoV2S1.IgG, CoV2S1S2.IgG, CoV2S2.IgG) compared with those who were managed on general medical floors (ward group). *p < 0.05; **p < 0.01. GRUs: Genalyte response units; ICU: Intensive care unit; Ig: Immunoglobulin.

There was a significantly lower IgM response to the nucleocapsid protein in the ICU versus the ward cohort (p = 0.0316). IgM response to the S protein subunits (including RBD) did not differ significantly between the ICU and ward groups (Figure 1A). However, IgG response against S1 and S2 subunits in the SARS-CoV-2 S protein was significantly higher in the ICU cohort (p = 0.0096 for S1; p = 0.0063 for S2; and p = 0.0064 for S1S2) (Figure 1B). IgG antibodies against the nucleocapsid and RBD antigens of SARS-CoV-2 were also increased in the ICU group but did not reach statistical significance (p = 0.0575 and 0.0650, respectively).

**Figure 2.**
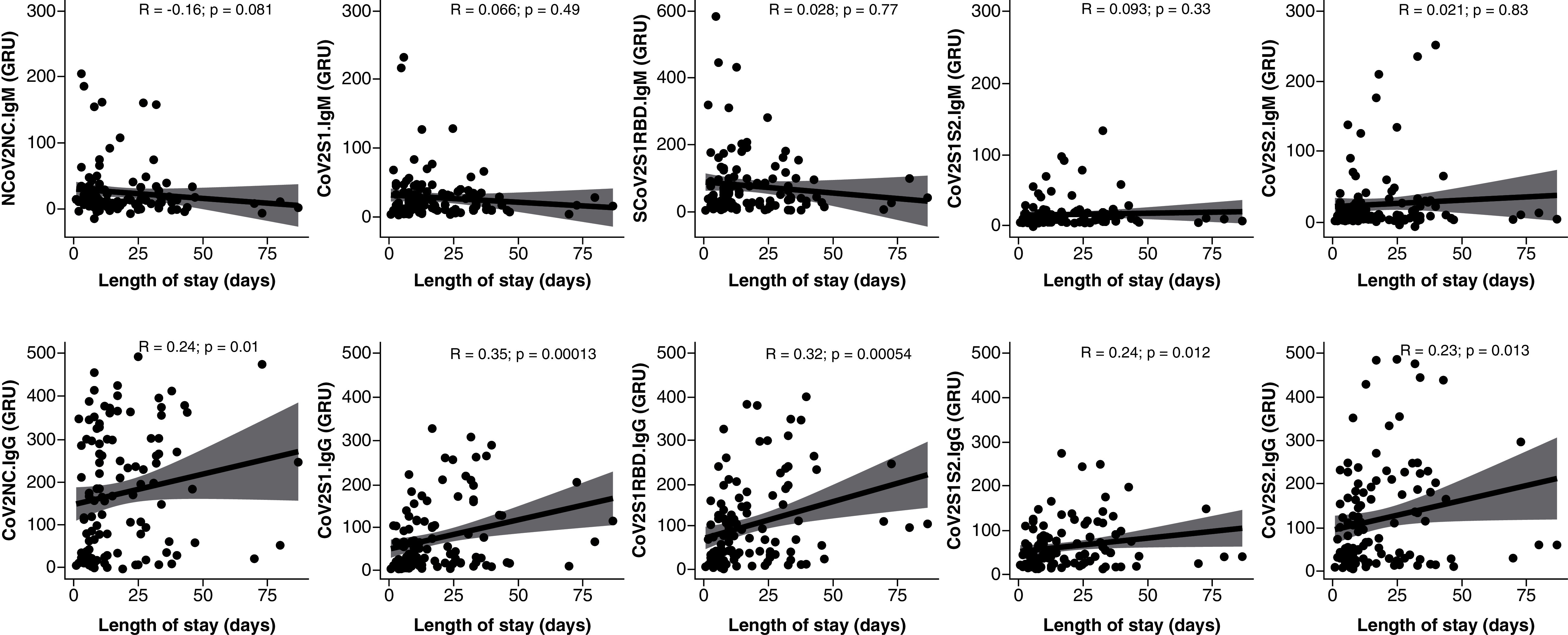
SARS-CoV-2 immunoglobulin G levels correlated with hospital length of stay. Correlation between antibody response was measured in GRUs to five SARS-CoV-2 epitopes and length of stay in hospitalized patients with COVID-19. IgM (top) and IgG (bottom) to five SARS-CoV-2 epitopes were measured. IgM was not significantly correlated with length of stay. IgG response to the nucleocapsid (CoV2NC.IgG) and all four S protein domains (CoV2S1.IgG, CoV2S1RBD.IgG, CoV2S1S2.IgG, CoV2S2.IgG) was positively correlated to length of stay. GRUs: Genalyte response units; Ig: Immunoglobulin.

Neither group showed cross reactivity to SARS-CoV-1, MERS or H1 antigens, and none of these antibody levels showed significant differences between ward and ICU patients (Supplementary Figure 1). Length of stay for all patients was positively correlated with IgG levels against the nucleocapsid (R: 0.24), S1 (R: 0.35), S1RBD (R: 0.32), S1S2 (R: 0.24) and S2 (R: 0.23), as seen in Figure 2. IgM was not correlated to length of stay (Figure 2). Additionally, there was no significant association between mortality during hospitalization and humoral response as assessed by the serology panel. Specifically, neither IgM nor IgG against any of the five components of the SARS-CoV-2 virus had a significant association in the 26 patients who died during their hospitalization.

## Discussion

This study aimed to assess the relationships among serology, disease severity and patient outcomes in hospitalized COVID-19 patients. As antibody testing becomes more prevalent, it is important to understand what serologic profiles may be expected in severe disease and if they can help guide management in the hospital. While several studies have revealed an association between severe disease and a higher IgG response to the SARS-CoV-2 S protein, others have shown either no association or lower IgG levels in those with severe symptoms [8–10]. In the present study, more severe disease requiring ICU care was associated with lower levels of IgM to SARS-CoV-2 nucleocapsid protein and higher levels of circulating IgG antibody to S protein at a mean of 9 days (median of 8 days) following symptom onset. This is in contrast to previous studies that have not seen a significant change at the same time point [8]. The present study is a large cohort with one of the most diverse epitope profiles studied to date, allowing for improved understanding of the humoral response to SARS-CoV-2 natural infection.

Early humoral antibody response to SARS-CoV-2, specifically lower IgM and higher IgG, may play a role in more severe disease or a more prolonged disease course, as seen in patients who require ICU level of care [5,6]. Anti-S IgG is detected at least 180 days after seroconversion and is the target for vaccination immunity because of its pivotal role in viral neutralization and disease prevention [11]. The present study's results show it is also an important correlate of disease severity and length of stay. A lower initial IgM antibody response may allow for increased levels of early viral replication and result in progression to severe disease [12]. The present study reinforces the association between severe infection requiring ICU admission and a higher early IgG response against multiple S protein regions [5,6]. This contrasts with some previous studies showing no significant increase in IgG levels during the same time intervals [10]. It remains unclear mechanistically why this is observed but may be due in part to higher viral load in those with severe disease, resulting in earlier and more robust activation of antibody production [13]. Alternatively, robust antibody response may be responsible for severe disease through antibody-dependent inflammation. Several studies have demonstrated an association between severe COVID-19 infection and increased production of S protein-specific IgG with altered glycosylation, specifically with decreased IgG1 fucosylation. Increased levels of these antibodies may enhance the production of pro-inflammatory cytokines and contribute to severe disease [14]. The present study's data confirm a positive correlation between IgG response to the nucleocapsid and the S proteins and length of stay.

The limitations of this study include most sample collection occurring at a median of 8 days post symptom onset, which had benefits in standardizing expected antibody response but did not allow longitudinal data evaluation. The authors did not assess for viral neutralization, making it difficult to theorize about whether the increased IgG response is a result or a cause of severe disease. Additionally, sample size and group similarity likely limited the ability to establish the significant associations between background characteristics and disease severity that are seen in other studies, including age, gender, obesity and diabetes [15,16].

Though higher levels of IgG were significantly associated with worse severity and longer hospital stay, overall mortality was not significantly correlated with any antibody response. This may be due to sample size, as only 26 patients died prior to discharge, and additional studies should evaluate IgG response and mortality in a larger population. As new variants of SARS-CoV-2 continue to evolve, understanding the patterns of anti-nucleocapsid IgM and anti-S IgG after native and/or breakthrough infection will be vital to understanding immunity and relative disease protection.

## Conclusion

In conclusion, robust IgG response, particularly against the spike protein, was associated with more severe disease from SARS-CoV-2, as indicated by increased need for ICU care and increased length of stay in the hospital. Conversely, severe disease was associated with lower IgM against the nucleocapsid protein. These differences in antibody response at a standard time interval (8 days post symptom onset) may be useful in triaging patients and predicting hospitalization needs in the future.

**Summary points**

**Introduction**

- The COVID-19 pandemic has caused significant morbidity and mortality and continues to drain resources among hospitals and healthcare professionals.
- Antibody testing has been developed that can assess levels of humoral immunity to multiple SARS-COV-2 proteins.

**Materials & methods**

- This retrospective analysis of sera from 113 hospitalized COVID-19 patients at 6–14 days (median: 8 days) post symptom onset correlated intensive care unit needs and length of stay with antibody titers.

**Results**

- Intensive care unit admission for patients with COVID-19 was associated with depressed anti-SARS-CoV-2 immunoglobulin (Ig)M response to the nucleocapsid protein and elevated anti-SARS-CoV-2 IgG response to the S protein.
- Length of stay was positively correlated with higher levels of IgG to both the S and the nucleocapsid proteins; IgM did not correlate with length of stay.

**Discussion**

- Patients requiring intensive care unit level of care have significantly higher IgG and lower IgM antibody responses than those hospitalized on the general medicine wards.
