# Supplementary figures and images for: Hospitalization requiring intensive care unit due to SARS-CoV-2 infection correlated with IgM depression and IgG elevation
Source: Future Sci OA. 2022 Feb 2;8(3):FSO783. doi: 10.2144/fsoa-2021-0126 (PMC8830355; doi:10.2144/fsoa-2021-0126)

Supplementary 1A

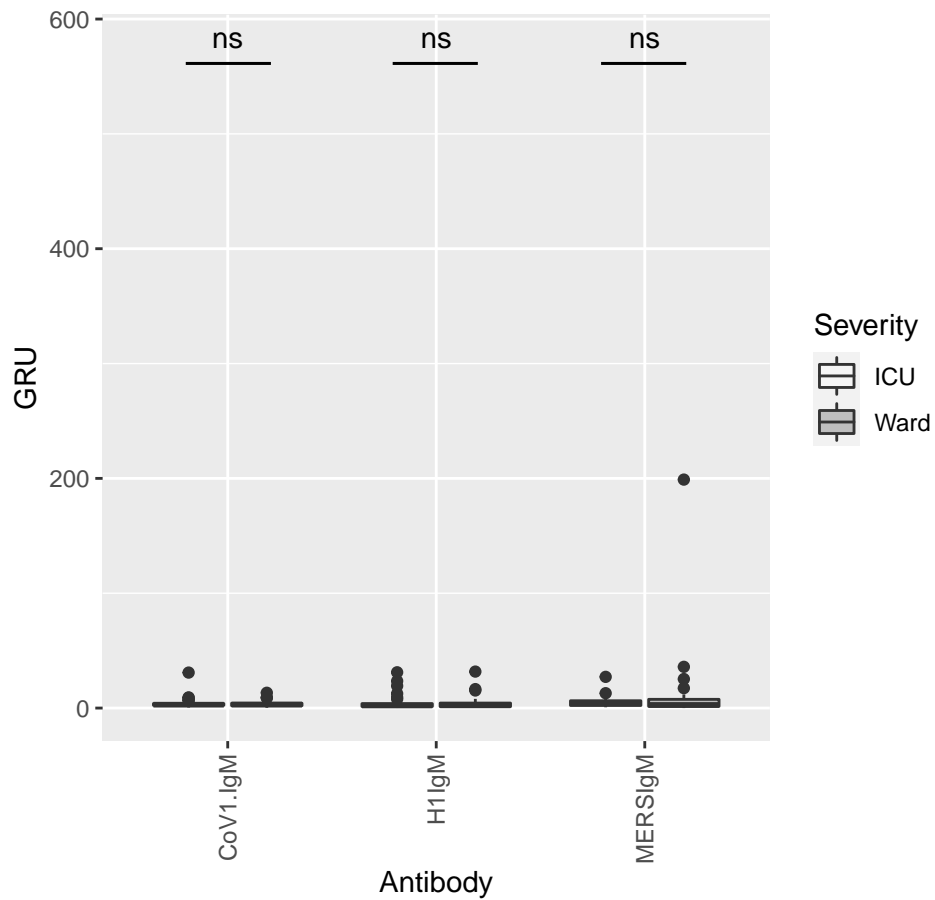

Supplementary 1B

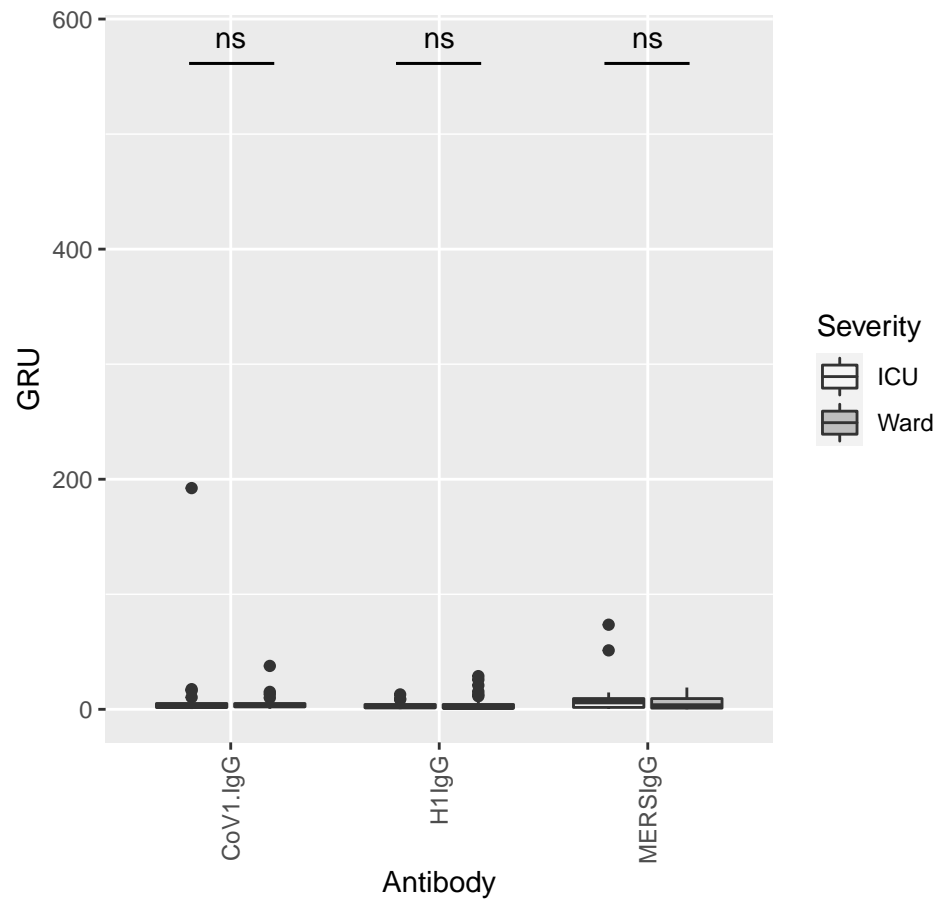

Supplement: Supplementary file 1 [file fsoa-08-783-s1.pdf]
